# Evaluation of Oxidative Stress Biomarkers in Patients with Fixed Orthodontic Appliances

**DOI:** 10.1155/2014/597892

**Published:** 2014-04-10

**Authors:** Sevil Sema Atuğ Özcan, İsmail Ceylan, Erkan Özcan, Nezahat Kurt, İlhan Metin Dağsuyu, Cenk Fatih Çanakçi

**Affiliations:** ^1^Faculty of Dentistry, Department of Orthodontics, Atatürk University, 25240 Erzurum, Turkey; ^2^Department of Periodontology, Oral Health Center, Gülhane Military Hospital, 06020 Ankara, Turkey; ^3^Department of Biochemstry, Faculty of Medical, Atatürk University, 25240 Erzurum, Turkey; ^4^Department of Orthodontics, Faculty of Dentistry, Osman Gazi University, 26140 Eskişehir, Turkey; ^5^Department of Periodontology, Faculty of Dentistry, Atatürk University, 25240 Erzurum, Turkey

## Abstract

*Aim.* The aim of this study was to examine the changes in the levels of interleukine-1 beta (IL-1**β**), tumor necrosis factor alpha (TNF-**α**), malondialdehyde (MDA), nitric oxide (NO), and 8-hydroxydeoxyguanosine (8-OHdG) in saliva and IL-1**β**, TNF-**α**, and NO in gingival crevicular fluid (GCF) samples of patients with fixed orthodontic appliances. *Material and Method.* The subject population consisted of 50 volunteers who were in need of orthodontic treatment with fixed orthodontic appliances. GCF and saliva samples were obtained from all individuals before treatment, at 1st month of treatment and at 6th month of treatment. Periodontal clinical parameters were measured. Samples were investigated to detect IL-1**β**, TNF-**α**, and 8-OHdG levels using ELISA method and NO and MDA levels using spectrophotometric method. *Results.* Since IL-1**β** level detected in GCF at the 6th month of orthodontic treatment is statistically significant according to baseline (*P* < 0.05), all other biochemical parameters detected both in saliva and in GCF did not show any significant change at any measurement periods. *Conclusion.* Orthodontic tooth movement and orthodontic materials used in orthodontic treatment do not lead to a change above the physiological limits that is suggestive of oxidative damage in both GCF and saliva.

## 1. Introduction

Orthodontic tooth movement (OTM) occurs as a result of controlled mechanical forces applied to teeth and biological response against these forces. Basic biological studies on orthodontic treatment are intended to understand the mechanisms including the conversion of the mechanical energy generated by orthodontic force to the biological reactions in the teeth and supporting tissues [1, 2]. These forces and mechanics ensure the desired tooth movement, whereas they can pose side effects, such as root resorption, gingival inflammation, attachment loss, and gingival retractions. The cellular and molecular response to the mechanical loading should be better understood in order to achieve desired tooth movement in a shorter time with fewer side effects for the tissues in orthodontic treatment [3].

A large number of inflammatory mediators called cytokines are involved in the biological response caused by the transfer of mechanical forces occurring in orthodontic treatment to the cells [4]. In the studies, cytokines have been reported to reflect the local microenvironmental status of periodontal tissues affected by orthodontic forces and to play a key role in the process of bone remodeling that is generated by orthodontic forces [3–6].

Inflammatory cells that play an important role in the production of cytokines migrate to the periodontal tissues especially through the dilated capillaries in the periodontal ligament during the tooth movement. Also, oxidative stress produced during orthodontic treatment may be associated with the expression of proinflammatory cytokines such as TNF-*α* and IL-1*β*. Reactive oxygen species (ROS) that causes oxidative damage includes both oxygen free radicals and nonradical oxygen derivates involved in oxygen radical production. The main ROS are hydrogen peroxide (H_2_O_2_), superoxide anion (O_2_^∙−^) and hydroxyl (OH^∙^) radicals. ROS potently affect adaptive immune maturation by promoting the synthesis of this proinflammatory cytokines by macrophages and dendritic cells via the mitogen activated protein kinase, activator protein-1, and nuclear factor kappa light chain enhancer of activated B cells [7, 8]. The studies on the pathogenesis of periodontal diseases have reported that a large number of ROS are released and stimulate the release of cytokines with the activation of inflammatory cells [3]. ROS can originate especially from PMNLs and monocytes, lymphocytes, platelets, osteoclasts, and even fibroblasts. The resulting ROS is considered as one of the most important factors of oxidative damage to tissues. It has been reported that DNA is exposed to oxidative damage 10^3^ times a day in every cell of the human body and that this is, however, a physiological event occurring also in healthy individuals due to the balance between DNA damage and repair [9, 10]. In orthodontic treatment, periodontium has an aseptic inflammation in which various cytokines are released by the effect of mechanical forces [4]. In the literature, no study has been reported on the oxidative damage that may occur as a result of aseptic inflammation in tissues with orthodontic tooth movements. We thus aimed to evaluate changes in the level of some oxidative stress markers by using GCF and saliva for determining oxidative damage that may occur in the process of remodeling, including periodontal tissue destruction caused by orthodontic tooth movement. NO is a free radical gas which acts as a physiological and pathophysiological mediator. In the present study NO was used to determine levels of ROS. ROS attack lipids, proteins, and DNA bases (purines and pyrimidines) and cause oxidative damage. This damage can be measured by various methods. MDA is a frequently used parameter in measuring the oxidative damage of the lipids resulting from free radicals. 8-OhdG is frequently used as a biomarker for DNA damage. For this purpose, we examined the changes in IL-1*β*, TNF-*α*, and NO levels to determine inflammatory activity in GCF and IL-1*β*, TNF-*α*, MDA, NO, and 8-OHdG in saliva, respectively, before treatment and at different stages of treatment.

## 2. Material and Methods

The subject population was consisted of a total of 50 patients, including 27 females and 23 males with ages ranging between 13 and 20 years, who applied to the Department of Orthodontics, Faculty of Dentistry, Ataturk University for therapeutic purposes. The study was approved by the same University's ethic board. Written informed consents of all patients was included in the study after giving verbal and written information about the purpose and method of the study. The study criteria is composed of having no systemic disease, having no medication use at the last six months, including antibiotics and anti-inflammatory drugs, having no substance abuse, being a nonsmoker, having a good cooperation and a good oral hygiene, being in the final phase of or completing the pubertal growth spurt, and being a candidate for fixed orthodontic treatment. The patients included in the study were given intermittent oral hygiene education before and during the study period. Clinical periodontal parameters were measured three times in total prior to orthodontic treatment and at the first and sixth months of orthodontic treatment. In addition, GCF and saliva samples were obtained. After the completion of the first pretreatment measurements, the nonextraction lower and upper fixed treatments have commenced in all patients.

The clinical examination was performed primarily in all patients participating in the study. We used Silness and Löe Plaque index (PI) and Löe and Silness Gingival index (GI) to evaluate the periodontal status in each patient and recorded probing pocket depth (PD) and clinical attachment loss (CAL).

### 2.1. Collection of GCF Samples

All clinical assessments except plaque index were conducted after collecting GCF samples in order not to affect GCF volume. Prior to the collection of GCF samples, we isolated the teeth with cotton rolls. In addition, we have investigated the presence of supragingival plaque on the tooth surface and removed these plaques with the help of cotton pellets. Air-water spray was positioned perpendicular to the tooth surface, and saliva or any blood was removed with aerial spraying for 5 seconds. Deep intracrevicular technique was used to obtain GCF samples from mesial and distal surfaces of maxillary incisors and canine teeth. Paper cones (standard periopaper) (ORAFLOW, Smithtown) were gently pushed into the gingival groove of GCF sampling teeth until any resistance was encountered and waited for 30 seconds. We did not use the cones contaminated with blood or saliva. These cones were studied in Periotron 8000 within 5 seconds to avoid evaporation after sampling, and the measured values obtained were converted to microliters by using PERİO.EXE software. The cones were places into Eppendorf tubes containing phosphate buffer solution after studying in Periotron 8000.

### 2.2. Collection of Saliva Samples

The saliva samples were collected according to the unstimulated saliva collection procedure. In order to avoid an interaction between any substance in the toothpaste and molecules to be examined, the patients were asked to brush their teeth without toothpaste and to add the saliva collected in their mouth into a tube. The collected samples are stored at −80°C until the biochemical analysis.

### 2.3. Biochemical Analysis of Saliva and GCF

In saliva and GCF samples, IL-1*β* and TNF-*α* analyses were made with ELISA method in accordance with the package inserts using DIAsource IL-1*β*-EASIA Kit (DIAsource ImmunoAssays, Louvain-la-Neuve, Belgium). In the saliva and GCF samples, nitrate/nitrite analysis was performed using Nitrate/Nitrite Colorimetric Assay Kit (Cayman Chemicals Company, Ellsworth Road, Ann Arbor) and in the saliva samples, TBARS Assay Kit (Cayman Chemical Company 1180 E. Ellsworth Road, Ann Arbor) was performed by using spectrophotometric method in accordance with the package inserts.

In our study, “Highly Sensitive 8-OHdG ELISA kit” was used in order to detect salivary 8-OHdG. Before analyzing saliva samples collected, they were shaken, transferred into 2 mL Eppendorf tubes, and centrifuged for 10 minutes. The resulting upper phase was transferred into another Eppendorf tube with the help of a graded micropipette and the amount of 8-OHdG was studied using 8-OHdG Elisa Kit (Highly Sensitive 8-OHdG Check, Japan Institute for the Control of Aging, Japan) in accordance with the package inserts.

### 2.4. Statistical Analysis

For all of the parameters used in our study, descriptive statistical analyzes comprising means and standard deviations were conducted separately in each registration period. A potential difference in the parameters between three different assessment periods was analyzed by Friedman test. As a result of these evaluations, we used Wilcoxon test to determine any significant difference in statistically significant parameters between the periods. Correlations between clinical parameters and laboratory parameters were examined by Spearman Rank Correlation Analysis. Analysis of the data was performed using MS-Excel and SPSS for Win. Ver. 20.00 (SPSS Inc., IL, USA) software packages. Statistical power analyzes were conducted using G Power version 3.1.3. A *P* value of <0.05 was considered as statistically significant.

## 3. Results

The mean age of a total of 50 patients, including 23 males and 27 females with ages ranging between 13 and 20 years, is 14.88 ± 1.47 years. Among patients treated with fixed orthodontic treatment, saliva and GCF samples were collected prior to orthodontic treatment and at the first and sixth months. The mean and standard deviation of biochemical parameters of saliva prior to the treatment and at the first and sixth months and the test result on the comparison of these periods to each other are shown in Table 1. Accordingly, it has been determined that there was no significant difference between the periods in terms of all the parameters measured in saliva (IL-1*β*, TNF-*α*, MDA, nitrite, and 8-OHdG) at different stages of the treatment (*P* > 0.05).

The mean and standard deviation of biochemical parameters of GCF prior to the treatment and at the first and sixth months and the test result on the comparison of these periods to each other are shown in Table 2. Therefore, we found no significant difference between the periods in terms of all the parameters measured in GCF except for IL-1*β* at different stages of the treatment (*P* > 0.05). We found a significant difference in IL-1*β* levels between the periods (*P* < 0.05).

The mean and standard deviations of the clinical periodontal parameters including probing pocket depth, plaque index and gingival index, and measurements of GCF volume prior to the treatment and at the first and sixth months, and Friedman test results on the comparison of these periods to each other are shown in Table 3. Accordingly, all clinical periodontal parameters showed a statistically significant differences before treatment and at different stages (*P* < 0.05), whereas no statistically significant difference was found in GCF volumes (*P* > 0.05). The clinical parameter CAL (Clinical attachment loss) was measured as 0.0 millimeters in all records of patients and thus was excluded from the statistical evaluations.

The results of Wilcoxon test to determine any significant difference between the periods in statistically significant parameters according to the Friedman test are shown in Table 4. Accordingly, in GCF measurements, a significant increase has been shown in IL-1*β* levels between pretreatment period and sixth month of treatment (*P* < 0.05) and in PI, GI, and PD values between pretreatment period and first month of treatment and pretreatment period and sixth month of treatment (*P* < 0.05).

## 4. Discussion

All the tissues of the body produce free radicals in physiological processes. The harmful effects of free radicals are counterbalanced by an antioxidant mechanism. A change in this balance in favor of free radicals deteriorates the oxidative balance and produces oxidative tissue damage associated with oxidative stress [10]. In the literature, there is no human study on the oxidative stress and oxidative damage that may occur as a result of aseptic inflammation in tissues with orthodontic tooth movements. Therefore, our study is the first on this issue.

The samples were taken three times including before treatment and at the first and sixth months of orthodontic treatment. There are many studies on evaluating the levels of cytokines like TNF-*α* and IL-1*β* in the early periods (such as 1 hour, 24 hours, and 7 days) of orthodontic tooth movement [11–13]. Unlike these studies, we aimed to investigate the long-term effects of orthodontic treatment on oxidative stress levels of oral environment. The correlation between the cytokine levels of TNF-*α* and IL-1*β* and periodontal health status is well known. Besides, many studies showed that periodontal disease leads to oxidative damage [14, 15]. In this study, we used cytokines as markers to determine whether periodontal diseases are present or not in molecular level, and also presence of oxidative damage as a result of periodontal disease has been evaluated. So, we did not measure cytokines levels in early stage of orthodontic tooth movement.

IL-1*β* is a cytokine with an important role in the osteoclast activation, bone resorption, and the biological response to the orthodontic forces [16]. TNF-*α* is a cytokine involved in bone resorption by being released from osteoblasts as well as monocytes and macrophages in acute and chronic inflammation [17] and under mechanical loading [11, 18]. In the studies, inflammatory cytokines such as IL-1*β* and TNF-*α* have also been reported to increase in GCF as a result of aseptic inflammation caused by orthodontic treatment [19, 20]. Many authors have reported that IL-1*β* and TNF-*α* levels reached the highest level at the early stage following orthodontic force [11–13]. These studies can be regarded as insufficient in evaluating changes in the level of cytokines at linear phase of orthodontic treatment [11], because these studies [12, 16] evaluating the effectiveness of cytokines in orthodontic tooth movement usually cover a period as short as a few months. Therefore, our study investigated changes in cytokine levels over a period of 6 months so as to include linear phase of treatment.

There are a limited number of studies evaluating the long-term changes in cytokine levels associated with the orthodontic tooth movement [19, 21]. Ren et al. [11], Başaran et al. [19] and Van Gastel et al. [21] have examined changes in the cytokine levels within the first three months, first 7 months and first year of orthodontic treatment, respectively. In these studies, cytokine levels have been reported to be maximized within first 24 hours and then approach baseline value. In accordance with these studies covering a long period, our study has also found no significant difference in IL-1*β* and TNF-*α* levels in the saliva at all measurement times, whereas it showed a substantial increase in IL-1*β* levels in GCF at sixth month compared to baseline. This result is compatible with the finding that, among these cytokines which are known to be involved in bone resorption and show a synergistic effect [22], IL-1*β* is more sensitive to mechanical stresses when compared with TNF-*α* [22].

The cytokines in GCF reflect changes resulting from periodontal inflammation and immune as well as biomechanical stress-related inflammatory reactions. Therefore, in compliance with Tzannetou et al.'s [23] study, we have included the patients with good oral hygiene in particular, and in a similar manner with Van Gastel et al.'s [21] and Ristic et al.'s [24] studies, we have continued an oral hygiene education throughout the treatment for the motivation of patients. However, in our study, PI, GI, and PD values were increased at different stages of treatment, whereas CAL levels were unchanged. Orthodontic materials such as bands, brackets, and arch-wires facilitate the accumulation of plaques, as well as adversely affect the removal of these plaques [25]. Thus, the formation of gingival inflammation is almost inevitable in patients undergoing fixed orthodontic treatment, although they have a very good motivation for oral hygiene [26, 27]. In our study, the increase in PI, GI, and PD values was found to be compatible with this situation; however, the measured values were within physiological limits in each stage of treatment.

There are different opinions about the change in the volume of GCF, which occurs in aseptic inflammation resulting from orthodontic tooth movements [28]. Some authors have emphasized that orthodontic forces cause an increase in GCF volume, whereas others advocate that they have no effect on GCF volume. Başaran et al. [19] have reported that the implementation of orthodontic forces increased the volume of GCF. Van Gastel et al. [21], in their study, have examined periodontal parameters at the beginning of orthodontic treatment at and 3 month after the removal of brackets and reported that GCF volume increased at the removal of the brackets compared with baseline and that this volume is still higher than baseline 3 months later. Drummond et al. [29] reported that GCF volume is not a reliable parameter reflecting orthodontic treatment-related tissue remodeling. In consistent with the studies indicating no change in GCF volume with orthodontic tooth movements [30, 31] we have found no significant change in GCF volume in all measurements prior to and at different stages of the treatment. Therefore, GCF volume alone may not be considered a reliable criterion in evaluating changes only due to orthodontic tooth movement independent of inflammation.

Another important finding of this study has is that NO levels did not change in all samples at any stage of treatment. Nilforoushan et al. [32] in their study on rats reported that NOS levels were considerably higher in the early stages of orthodontic tooth movement and reached the highest level between 24th and 48th hours and again returned to baseline during the course of treatment. D'Atillio et al. [33] reported that NOS levels were higher in the early stages of thetreatment (in the 2nd week) in patients undergoing orthodontic treatment. These studies which cover a short period of time from several days to several weeks, imlicate decrease in NO levels to baseline at the end. Although our study is a long-term study, our results can be considered to be compatible with these studies in terms of lack of significant change in NO levels during the later stages of treatment. In the present study, the lack of a significant change in NO levels in the first and sixth months of orthodontic treatment can be interpreted as that the level of periodontal free radicals remained within physiological limits as a result of the tooth movements provided by orthodontic treatment and the materials used for the treatment.

8-OHdG is a molecule which is a recently popular molecule in determining oxidative damage also in the periodontal inflammation. It attracts attention as a widely used molecule in the evaluation of oxidative damage that may occur especially in periodontal tissues [9, 34]. However, we have not revealed any literature studies investigating oxidative DNA damage that can arise from aseptic inflammation due to the orthodontic treatment. In our study, salivary 8-OHdG levels measured in different periods of the treatment displayed no significant change in the course of this treatment. Based on this result, orthodontic tooth movement and the aseptic inflammation induced by the treatment can be said not to cause oxidative damage in the DNA of the periodontium. In addition, the lack a significant change in 8-OHdG levels which is known to increase in periodontal inflammation can be considered as an evidence of the absence of significant periodontal damage in our subjects that underwent orthodontic treatment.

Buljan et al. [35] in their in vitro studies investigating 8-OHdG levels as a marker of oxidative stress reported that different types of brackets could be a source of oxidative stress due to their substance elements such as iron and nickel, and however full metal and polymeric brackets generate a greater oxidative stress. Faccioni et al. [36] have detected a DNA damage in buccal mucosa because of the nickel and cobalt released from the brackets and arch-wires. Takane et al. [37] reported that the metal ions released by orthodontic materials can be identified in saliva, blood, and urine, albeit not in toxic concentrations. In parallel with these studies, the lack of a significant change in salivary levels of 8-OHdG in the treatment process supports the idea that full metal brackets, tubes, and wires are not toxic so as to cause an oxidative DNA damage.

The lipids are one of the most damaged cellular components by reactive oxygen species. The oxidative stress caused by the deterioration of the balance between oxidants and antioxidants leads to the lipid peroxidation [38]. The last product of the lipid peroxidation process, malondialdehyde (MDA), is a frequently used parameter in measuring the oxidative damage to the lipids resulting from free radicals [39]. Thiobarbituric acid reactive substances test (TBARS) is the most widely used test in the measurement of MDA levels [40, 41]. Some previous studies have shown that serum levels of MDA were increased in systemic diseases such as tuberculosis and that salivary levels of MDA were also significantly increased in periodontal diseases and reported that this increase in MDA levels is an important indicator of periodontal tissue destruction [38]. The analysis of salivary levels of MDA is also of importance as it gives an idea about the general periodontal health. Khalili and Biloklytska [39] in their study examining MDA levels in saliva in healthy subjects and patients with mild, moderate, and severe periodontitis reported that MDA levels in healthy individuals were lower than that of patients with periodontitis. In the present study, no significant difference was found in the salivary levels of MDA measured in different treatment periods. Based on this result, orthodontic forces and the therapeutic materials can be said not to cause an oxidative damage to the lipid components of tissues in the first six months of the treatment. This can be considered as an indicator of the lack of periodontal inflammation in patients receiving orthodontic treatment.

In conclusion, orthodontic tooth movement and orthodontic materials used in orthodontic treatment do not lead to a change above the physiological limits that is suggestive of oxidative damage in both GCF and saliva.

## Figures and Tables

**Table 1:** Biochemical parameters of saliva prior to the treatment and at the first and sixth months.

SALIVA	*N*	Pretreatment	Posttreatment 1st month	Posttreatment 6th month	*P* value
Parameters		Mean ± std.	Mean ± std.	Mean ± std.	
IL-1*β*	50	40.80 ± 41.6	51.86 ± 61.20	40.98 ± 33.56	0.474
TNF-*α*	50	18.99 ± 14.31	25.58 ± 25.66	21.18 ± 18.78	0.256
MDA	50	3.76 ± 2.48	3.87 ± 3.06	3.60 ± 2.45	0.984
Nitrite	50	4.58 ± 3.97	4.47 ± 4.25	3.78 ± 3.29	0.565
8-OHdG	50	4.73 ± 4.15	5.38 ± 5.77	4.64 ± 4.03	0.942

**Table 2:** Biochemical parameters of GCF prior to the treatment and at the first and sixth months.

GCF	*N*	Pretreatment	Posttreatment 1st month	Posttreatment 6th month	*P* value
Parameters		Mean ± std.	Mean ± std.	Mean ± std.	
IL-1*β*	50	10.69 ± 5.11	11.99 ± 6.74	13.41 ± 6.74	0.041*
TNF-*α*	50	6.83 ± 9.58	6.66 ± 3.80	6.81 ± 3.96	0.254
Nitrite	50	3.18 ± 1.71	3.56 ± 1.70	3.62 ± 2.07	0.096

*P < 0.05.

**Table 3:** Clinical periodontal parameters measurements of GCF volume prior to the treatment and at the first and sixth months.

Clinical parameters	*N*	Pretreatment	Posttreatment 1st month	Posttreatment 6th month	*P* value
		Mean ± Std.	Mean ± Std.	Mean ± Std.	
PI	50	0.12 ± 0.14	0.31 ± 0.28	0.20 ± 0.30	0.000*
GI	50	0.07 ± 0.08	0.27 ± 0.28	0.31 ± 0.30	0.000*
PD	50	1.48 ± 0.30	1.64 ± 0.30	1.73 ± 0.33	0.000*
GCF volume	50	0.07 ± 0.02	0.08 ± 0.03	0.09 ± 0.07	0.136

*P < 0.05.

**Table 4:** The results of Wilcoxon test to determine any significant difference between the periods in statistically significant parameters according to the Friedman test.

	Wilcoxon signed ranks test					
	Pretreatment—1st month		Treatment 1st–6th month		Pretreatment—6th month	
	*Z*	Sig.	*Z*	Sig.	*Z*	Sig.
IL-1*β* (GCF)	−1.78	0.074	−1.34	0.181	−3.05	0.002**
PI	−4.70	0.000***	−0.056	0.955	−4.41	0.000***
GI	−5.16	0.000***	−1.27	0.203	−5.48	0.000***
PD	−3.79	0.000***	−2.14	0.032*	−4.61	0.000***

*P < 0.05,  **P < 0.01,  ***P < 0.001.
